# Interaction between the α-glucosidases, sucrase-isomaltase and maltase-glucoamylase, in human intestinal brush border membranes and its potential impact on disaccharide digestion

**DOI:** 10.3389/fmolb.2023.1160860

**Published:** 2023-03-08

**Authors:** Stephanie Tannous, Tammy Stellbrinck, Abdullah Hoter, Hassan Y. Naim

**Affiliations:** Department of Biochemistry, University of Veterinary Medicine Hannover, Hannover, Germany

**Keywords:** sucrase-isomaltase, maltase-glucoamylase, human brush border membrane, protein-protein interaction, digestive function, carbohydrates

## Abstract

The two major intestinal α-glycosidases, sucrase-isomaltase (SI) and maltase-glucoamylase (MGAM), are active towards α-1,4 glycosidic linkages that prevail in starch. These enzymes share striking structural similarities and follow similar biosynthetic pathways. It has been hypothesized that starch digestion can be modulated *via* “toggling” of activities of these mucosal α-glycosidases, suggesting a possible interaction between these two enzyme complexes in the intestinal brush border membrane (BBM). Here, the potential interaction between SI and MGAM was investigated in solubilized BBMs utilizing reciprocal pull down assays, i.e., immunoprecipitation with anti-SI antibody followed by Western blotting with anti-MGAM antibody and *vice versa*. Our results demonstrate that SI interacts avidly with MGAM concomitant with a hetero-complex assembly in the BBMs. This interaction is resistant to detergents, such as Triton X-100 or Triton X-100 in combination with sodium deoxycholate. By contrast, inclusion of sodium deoxycholate into the solubilization buffer reduces the enzymatic activities towards sucrose and maltose substantially, most likely due to alterations in the quaternary structure of either enzyme. In view of their interaction, SI and MGAM regulate the final steps in starch digestion in the intestine, whereby SI assumes the major role by virtue of its predominant expression in the intestinal BBMs, while MGAM acts in auxiliary supportive fashion. These findings will help understand the pathophysiology of carbohydrate malabsorption in functional gastrointestinal disorders, particularly in irritable bowel syndrome, in which gene variants of SI are implicated.

## 1 Introduction

Starch digestion is a process that implicates salivary and pancreatic α-amylases and the two major intestinal α-glycosidases, sucrase-isomaltase (SI, EC 3.2.148 and 3.2.1.10) and maltase-glucoamylase (MGAM¸ EC 3.2.1.20 and 3.2.1.3) (Conklin et al., 1975; Husein et al., 2020). The hydrolytic functions of SI and MGAM are responsible for almost all carbohydrates that are linked *via* α-1,2, α-1,4 and α-1,6 linkages and comprise the majority of the typical diet in humans. SI is capable of digesting all the aforementioned linkages, while MGAM cleaves mainly α-1,4 linkages, the major glycosidic linkage in starchy food, and to a lesser extent also α-1,2, α-1,3, and α-1,6 glycosidic linkages (Lee et al., 2016). With almost 80% digestive capacity of α-1,4 glycosidic linkages, SI has been proposed to contribute substantially to mucosal MGAM activity (Quezada-Calvillo et al., 2007b; Diaz-Sotomayor et al., 2013). SI is also exclusively responsible for sucrose (α-D-glucopyranosyl-(1→2)-β-D-fructofuranoside; SUC) digestion and all isomaltase (1,6-O-α-d-glucanohydrolase; IM) activity (Lin et al., 2012). The complementing activities of SI and MGAM are required for the mucosal digestion of α-D-glucose oligomers that originate from plants (Lin et al., 2012).

The genes encoding SI and MGAM are located on chromosome 3 (3q26.1) and chromosome 7 (7q34) respectively (Nichols et al., 2003). SI and MGAM are members of the glycosyl hydrolase family 31 (GH31), with a remarkable 58% identity in their amino acid sequence (Nichols et al., 1998). SI and MGAM are type 2 membrane-bound glycoproteins that are efficiently expressed at the apical or microvillus membrane of the enterocytes (Nichols et al., 2003). The α-glucosidase activities of SI and MGAM are located within the WI**D**MNE motif that corresponds to the consensus pattern of [GFY]-[LIVMF]-W-x-**D**-M-[NSA]-E for the signature I of the GH31 family, whereby the aspartic acid residue represents the nucleophile. Further catalytic domain, HWLG**D**N, comprises another aspartic acid as a putative proton donor in all four subunits of SI and MGAM and is located in a conserved sequence within exons 17 and 40 (Gericke et al., 2017b). SI is exclusively expressed in enterocytes, while several forms of MGAM in various tissues exist. In the enterocytes, two related isoforms of MGAM that share several repeats have been recorded in NCBI database. One of these isoforms has been cloned with comparable exon-intron arrangements to the SI gene and the deduced protein sequences, 1857 amino acids, share 58.4% identity and 74.3% similarity based on global pairwise sequence alignment analysis (EMBOSS Needle (Li et al., 2015)) (Nichols et al., 2003). A larger sequence, predicted by automated computational analysis derived from a genomic sequence (NC_000007.14), encodes 2,753 amino acids and has been cloned in our laboratory (Hoter and Naim, unpublished data). Likewise, comparison of the tertiary structures resolved for the N-termini of SI and MGAM revealed overlying protein domains (Sim et al., 2010). The larger version of MGAM is the dominant intestinal form of an apparent molecular weight of 335 kDa. This has been identified in intestinal biopsy specimens by immunoprecipitation with specific monoclonal antibodies (mAbs) against MGAM (Naim et al., 1988a). Upon maturation in the enterocytes and proper sorting to the apical membrane, SI is cleaved in the intestinal lumen by pancreatic trypsin to its two subunits sucrase (SUC) and isomaltase (IM), which remain associated with each other *via* strong ionic interactions (Jacob et al., 2000b; Gericke et al., 2017b). Western blotting of intestinal homogenates reveals mainly SUC, IM, and the uncleaved SI precursor, while in biosynthetic labeling experiments, only uncleaved SI is immunoprecipitated since trypsin is not present in the culture medium of biopsy specimens (Jacob et al., 2000b). MGAM behaves in a similar manner and only the precursor is revealed in biosynthetically labeled biopsies. Cleavage of the MGAM precursor in the enterocytes in a fashion similar to SI has been also reported for many species, but not for the human enzyme (Naim et al., 1988b).

Recently we have shown that SI and MGAM in combination comprise about 11% of total intestinal brush border membrane (BBM) proteins, whereby SI expression level is almost 3-folds higher than that of MGAM (Amiri and Naim, 2017). Of note the specific activity of MGAM towards α-1,4-glyosidically linked disaccharides, the most common linkage in disaccharides, is almost 3-folds higher than that of SI (Ao et al., 2007). However, it is not elucidated until present whether the two enzymes SI and MGAM by virtue of their striking structural similarities and biosynthetic pathways interact with each other and may modulate each other’s activities towards α-1,4-glycosidically linked disaccharides. *In vitro* studies with recombinant forms of the individual subunits of SI and MGAM have proposed a modulation of starch digestion for slow glucose release through possible “toggling” of activities of mucosal α-glucosidases (Lee et al., 2012). This mechanism suggested an interaction between the two enzyme complexes that may occur in close proximity to each other. The elucidation of a functional and physical interaction of SI and MGAM is critical in the context of understanding the pathophysiology of carbohydrate malabsorption in functional gastrointestinal disorders (FGIDs), such as congenital sucrase-isomaltase deficiency (CSID) or irritable bowel syndrome (IBS). In this study, we have analyzed the interaction between the two proteins in human intestinal BBMs and assessed the implication of this interaction on their function.

## 2 Materials and methods

### 2.1 Reagents

Tissue culture plates were bought from Sarstedt (Nüembrecht, Germany). DEAE-dextran, trypsin-EDTA, fetal calf serum for cell culture, Dulbecco’s Modified Eagle’s Medium (DMEM), penicillin, streptomycin, protease inhibitors, ProtA-Sepharose, trypsin, Triton X-100, and deoxycholate were purchased from Sigma- Aldrich (Deisenhofen, Germany). Acrylamide, TEMED, Tris, SDS, dithiothreitol (DTT), polyvinyl difluoride (PVDF) membrane, as well as sucrose, maltose, and palatinose were purchased from Carl Roth GmbH (Karlsruhe, Germany). Glucose oxidase-peroxidase mono-reagent was bought from Axiom GmbH (Bürstadt, Germany). Molecular weight standards for SDS-PAGE SuperSignal™ West Fermento maximum sensitivity Western blot chemiluminescence substrate were purchased from Thermo Fisher Scientific GmbH (Schwerte, Germany).

### 2.2 Cell lines, tissues and antibodies

Monkey kidney COS-1 cells (ATCC CRL-1650) were maintained in DMEM medium at 37°C in a humidified atmosphere and 5% CO_2_. Human intestinal BBMs were a generous donation to our laboratory made by the late Dr. Erwin Sterchi, University of Bern, Switzerland. The BBMs were approved by the ethical committee at the University of Bern. These membranes were prepared from the jejunum in the Sterchi laboratory using the modified divalent cation precipitation method, according to Schmitz et al. (1973) and as modified by Sterchi and Woodley (1980). Specific monoclonal mouse antibodies against SI were used: HBB 2/614/88 (anti-SUC), HBB 3/705/60 (anti-IM), HBB 2/219/20 (anti-SI) (Hauri et al., 1985b), hSI2 (anti-SI) (Beaulieu et al., 1989). Similarly, specific monoclonal mouse antibodies against MGAM were used: HBB 3/41, HBB 4/46/5/1, LAMA 1/207/140/12, LAMA 1/77/6/2/1, LAMA 1/127 (Hauri et al., 1985b; Nichols et al., 2003; Quezada-Calvillo et al., 2007a; Amiri and Naim, 2017). The anti-SI and anti-MGAM antibodies were generously provided by Dr. Hans-Peter Hauri (Bern, Switzerland) (Hauri et al., 1985b), and Dr. Buford L Nichols (Baylor College of Medicine, Texas) (Beaulieu et al., 1989).

### 2.3 Plasmids

The cDNA encoding wild type SI or variants of SI are routinely used in our laboratory (Gericke et al., 2017b; Husein et al., 2020). A 2753 base pairs long cDNA encoding the predominant 335 kDa long form of intestinal MGAM has been generated based on the published MGAM NCBI sequence (NM_001365693.1) and using a template 1857 pairs long cDNA encoding the short version of MGAM that was kindly provided by Dr. Nichols, Baylor College of Medicine, Houston, United States (Nichols et al., 1998).

### 2.4 Cell transfection with cDNA encoding SI and MGAM, immunoprecipitations and western blotting

COS-1 cells were transiently transfected with cDNA encoding either SI or MGAM using the diethylaminoethyl (DEAE)-dextran method (Naim et al., 1991). After transfection the cells were washed twice with phosphate buffered saline (PBS) (pH 7.4) before solubilization using a buffer containing 25 mM Tris-HCl, pH 8.0, 50 mM NaCl, 0.5% sodium deoxycholate (DOC) and 0.5% Triton X-100 (TX-100). SI and MGAM were immunoprecipitated using anti-SI mAbs, hSI2, HBB 2/219, and HBB 2/614, or anti-MGAM mAbs, HBB 3/41 and HBB 4/46 (Hauri et al., 1985b; Nichols et al., 2003; Quezada-Calvillo et al., 2007a); the immunoprecipitates were processed for SDS-PAGE on 6% slab gels followed by Western blotting according to routinely utilized procedures in our laboratory (Alfalah et al., 2009; Gericke et al., 2017a; Henström et al., 2018; Husein and Naim, 2020). HBB 2/614, anti-SUC, HBB 3/705, anti-IM or a combination of mAbs that recognize MGAM (77/127/207) followed by HRP-conjugated secondary antibody (Thermo Fischer, Schwerte, Germany) were used for Western blotting. The chemiluminescence signals were detected using the ChemiDoc MP™ Touch Imaging System (Bio-Rad, Munich, Germany).

### 2.5 Solubilization of human brush border membranes, co-immunoprecipitations with anti-SI and anti-MGAM antibodies and enzymatic activity measurements

Highly purified BBMs (15 μg/mL) were solubilized in a buffer containing 10 mM Tris-HCl, pH 7.4, and 150 mM NaCl with 1% TX-100 (referred to thereafter as TX-100) or 25 mM Tris-HCl, pH 8.0, and 50 mM NaCl with 0.5% DOC/0.5% TX-100 (referred to later as DOC/TX-100). The samples were processed for immunoprecipitation with anti-SI (hSI2, HBB 2/219, and HBB 2/614) or anti-MGAM (HBB 4/46) and resolved by SDS-PAGE (6% slab gels) followed by Western blotting using anti-MGAM (77/127/207), anti-IM (HBB 3/705) or anti-SUC (HBB 2/614) antibodies to verify the interaction between the two molecules.

The enzymatic activities of SI and MGAM were assessed essentially according to Dahlqvist (1970). Here again, 15 μg/mL BBMs were solubilized with 1% TX-100 or 0.5% TX-100/0.5% DOC in 10 mM Tris-HCl, pH 6.1, and 150 mM NaCl. For determination of sucrase activity 50 µL of these solubilized BBMs were incubated with 50 µL of 56 mM sucrose. Similarly, maltase activity was measured following diluting 10 µL of solubilized BBMs in 40 µL buffer containing 10 mM Tris-HCl, pH 6.1, and 150 mM NaCl and subsequent incubation with 50 µL of 56 mM maltose for 1 h at 37°C. Either sucrose or maltose substrate was dissolved in a buffer containing 10 mM Tris-HCl, pH 6.1 and 150 mM NaCl. The liberated glucose was determined by adding 900 µL glucose fluid (GOD-PAP) (Axiom Diagnostics, Florida, United States) and incubating for 20 min at 37°C and undergoing photometric measurement at 492 nm using the Plate Reader Epoch (BioTek). In another set of experiments the enzyme kinetics were determined. For this, the BBM lysates, solubilized with TX-100 or 0.5% TX-100/0.5% DOC as described above, were incubated with different concentrations of sucrose or maltose (0 mM–60 mM) for 1 h at 37°C, and the K_m_ values were assessed by Michaelis-Menten constants.

### 2.6 Statistical analysis

Detection and quantification of the protein bands were achieved using Image Lab (Bio-Rad Laboratories GmbH, Munich, Germany). All calculations were performed in Microsoft Excel. Data represent the results obtained from at least three independent repeats and the reported error bars represent the standard error of the mean (SEM). Statistical analysis, unpaired and paired t-tests and non-linear regression were performed using GraphPad Prism with the statistical significance set at * *p* < 0.05, ** *p* < 0.005, and *** *p* < 0.0005.

## 3 Results

### 3.1 Assessment of the specificities of the antibodies directed against SI and MGAM

**FIGURE 1:**
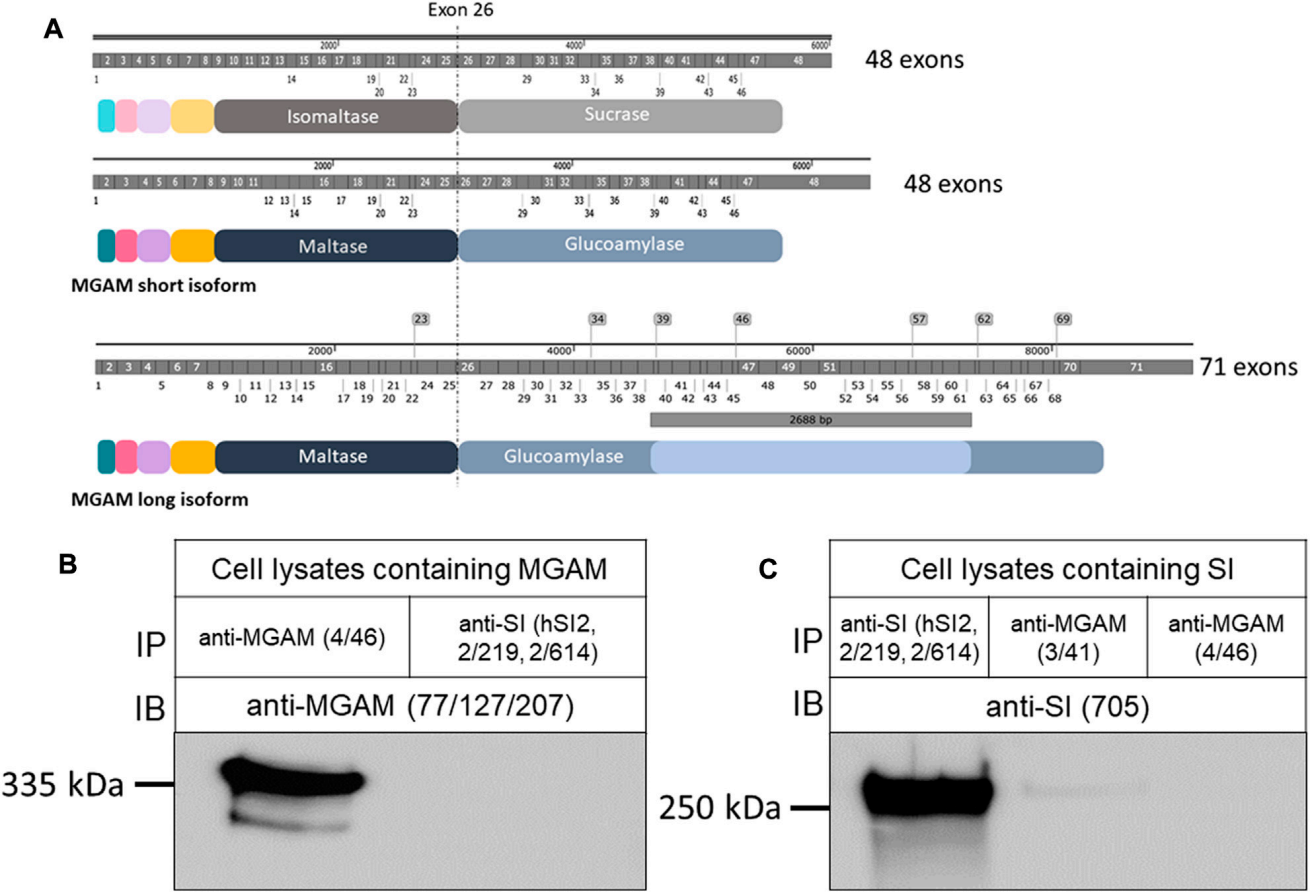
Structural features and protein characterization of SI and MGAM. **(A)** mRNA**,** protein domains and structural similarities between SI and MGAM isoforms. Both SI and the short isoform of MGAM (NCBI sequence NM_004668.3) comprize 48 exons while the longer intestinal isoform (NCBI sequence NM_001365693.1) contains additional 2,688 bp that span the region between exons 38 and 61 as represented in the illustration. At the protein level, SI is composed of 1827 amino acids while the short isoform of MGAM has 1857 amino acids. The longer MGAM isoform that is found mainly in the intestine contains 2,753 amino acids. Notably, the position and length of exons in SI and MGAM reveal high similarity. **(B)** MGAM or **(C)** SI were expressed in COS-1 cells and the detergent extracts of these cells were immunoprecipitated with either anti-SI (hSI2, HBB 2/219, and HBB 2/614) or anti-MGAM (HBB 3/41 or HBB 4/46) antibodies, and immunoblotted or reciprocally immunoblotted using either anti-SI (HBB 3/705) or anti-MGAM (77/127/207) antibodies. Western blot revealed specific protein bands in the left lanes and no bands in the right lanes of both blots confirming the specificity and lack of cross reactivity between the antibodies used.

In view of the striking sequence homologies between SI and MGAM (Figure 1A), cross-reactivity of mAbs against either protein cannot be excluded. In our previous studies, immunoprecipitation of SI was performed with the mAb, HBB 2/219 that is directed to separate epitopes of these proteins and recognize native conformation as well as potential conformational changes of SI in normal intestinal biopsy specimens and also in CSID. The immunoprecipitations of MGAM utilized the HBB 4/46 mAb. Initial screening of these mAbs ruled out potential cross-reactivity (Hauri et al., 1985a).

We first confirmed these data by individual expression of SI or MGAM in COS-1 cells, which do not express either protein, followed by reciprocal immunoprecipitations of the expressed proteins with mAbs anti-SI and Western blotting with anti-MGAM antibodies or *vice versa* (Figures 1B,C). The controls comprised immunoprecipitations of the expressed proteins with their own specific mAbs. It should be noted that expression of SI in COS-1 cells or other cell lines, such as Caco-2 cells, reveals the full length uncleaved precursor SI, due to the absence of pancreatic trypsin in the culture medium. As shown in Figure 1C, immunoprecipitation of SI from detergent extracts of transfected COS-1 cells using a combination of several mAbs against SI followed by Western blotting with the mAb HBB 3/705, which is directed against IM as well as the SI precursor, revealed a 245–250 kDa band corresponding to SI. Immunoprecipitations of similar detergent extracts with mAb HBB 3/41 or HBB 4/46, both are directed towards MGAM (Amiri and Naim, 2017), did not pull down SI. Nevertheless, due to the detection of a very faint minor band with mAb HBB 3/41, we excluded this antibody from our further analyses to avoid potential antibody cross reactivity and carried out further immunoprecipitations of MGAM with mAb HBB 4/46. The reciprocal experiment using detergent extracts of cells expressing MGAM revealed a 335 kDa protein band corresponding to MGAM that was pulled down by its own antibody (HBB 4/46) and not anti-SI mAbs. These data unequivocally demonstrate that there is no cross-reactivity between mAb HBB 4/46 (anti-MGAM) and the anti-SI mAbs, hSI2, HBB 2/219, and HBB 2/614. Therefore, these antibodies have been used further to assess potential interactions between SI and MGAM.

### 3.2 Pull-down experiments reveal interacting SI and MGAM molecules under different solubilization conditions

**FIGURE 2:**
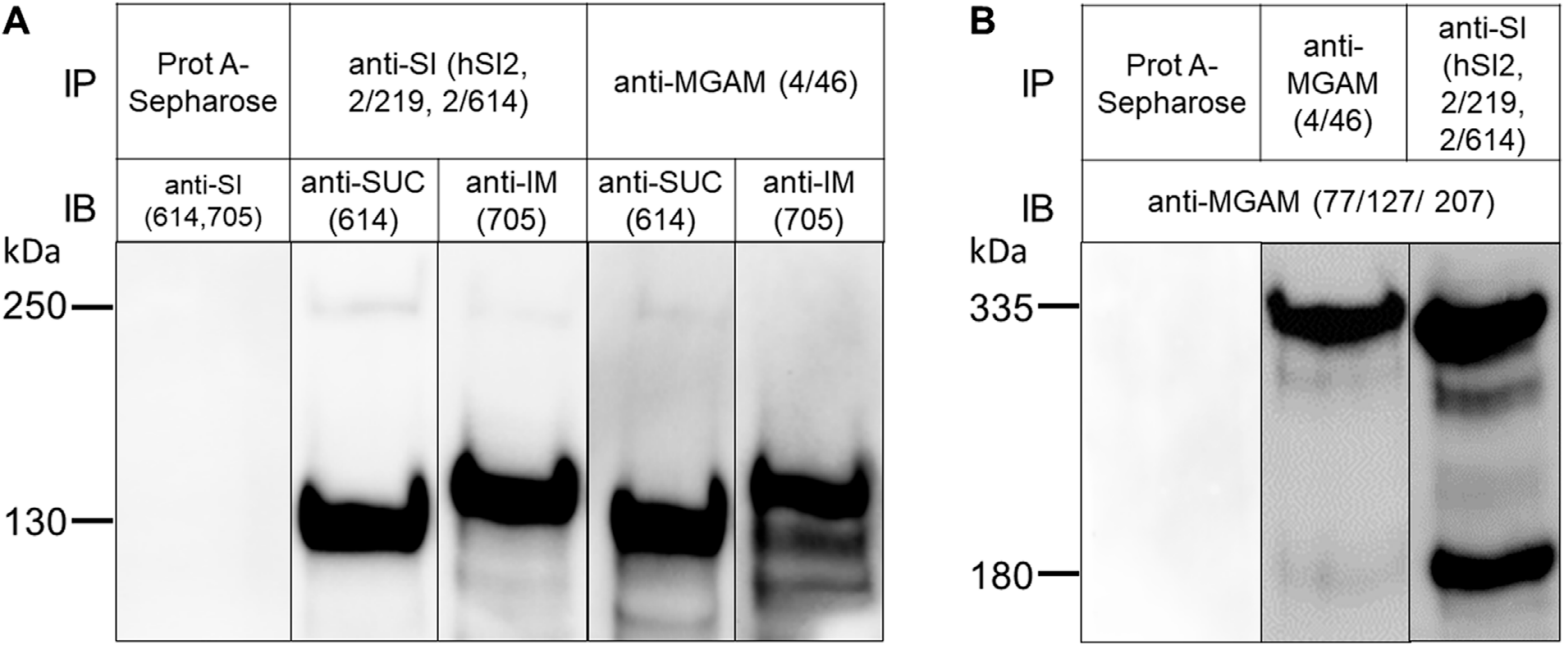
Sucrase and isomaltase interact avidly MGAM in TX-100-solubilized human brush border membranes. **(A)** BBMs of human jejunum were solubilized with 1% TX-100 in a buffer containing 10 mM Tris-HCl, pH 7.4, 150 mM NaCl, and immunoprecipitated with either anti-SI (hSI2, HBB 2/219, HBB 2/614) or anti-MGAM (HBB 4/46) antibodies, or without antibodies to exclude any unspecific binding to Protein A-Sepharose (first lane). Western blot of the immunoprecipitated proteins using anti-SI antibodies revealed two specific bands corresponding to sucrase (SUC) and isomaltase (IM) domains of SI protein, respectively and regardless of the anti-SI or anti-MGAM antibodies used for immunoprecipitation. **(B)** BBMs were solubilized and immunoprecipitated as described in **(A)**. Immunoblotting of the immunoprecipitated proteins revealed MGAM protein band at M.W of 335 kDa in the two lanes irrespective of the anti-SI or anti-MGAM antibodies used for immunoprecipitation. These data clearly demonstrate co-immunoprecipitation and interaction of SUC and IM with MGAM and *vice versa* under the buffer conditions described. SUC and IM were recognized by HBB 2/614 (anti-SUC) and HBB 3/705 (anti-IM) respectively and MGAM was recognized by anti-MGAM antibodies (77/127/207). IP, immunoprecipitation; IB, immunoblotting.

Human BBM preparations were used for assessing the interaction between SI and MGAM. These membranes have been initially isolated from the jejunum of the human intestine of a kidney donor using the CaCl_2_ divalent cation procedure (Schmitz et al., 1973). First, the contents of SI and MGAM in these membranes were verified at the functional level by assessment of the activities towards sucrose, palatinose, and maltose, which were 2.3 ± 0.6 IU/mg for SUC, 0.3 ± 0.002 IU/mg for IM, and 19.4 ± 8.3 IU/mg for both MGAM and SUC due to the joint digestive function of both enzymes towards α-1,4 glycosidic linkage of maltose (Quezada-Calvillo et al., 2007b; Nichols et al., 2017). Next, we assessed the expression of SUC, IM and MGAM in these membranes at the protein level by immunoprecipitation of detergent extracts of these membranes with mAbs anti-SI and anti-MGAM followed by Western blot analysis. The negative control employed the addition of BBM lysates to Protein A-Sepharose beads without prior addition of either anti-SI or anti-MGAM antibodies, which expectedly showed no binding of SI or MGAM to Protein A-Sepharose (Figures 2A,B). Figure 2A shows the immunoprecipitated forms of SUC and IM that have been recognized by mAbs HBB 2/614 and HBB 3/705 respectively (Naim et al., 1991; Amiri and Naim, 2017). Both antibodies detected also a faint protein band corresponding to the uncleaved SI precursor confirming previous results (Hauri et al., 1985a). In Figure 2B, the mAbs anti-MGAM (77/127/207) used for the Western blots recognized predominantly an uncleaved protein band of 335 kDa (Hauri et al., 1985b) and also a definite band of 180 kDa that corresponds likely to a cleaved form of MGAM. The detection of this band in the purified jejunal BBMs indicates that a partial cleavage of the MGAM precursor occurs in the intestinal lumen at the apical membrane of the enterocytes. This form has not been detected before in biosynthetic labeling of biopsy samples that revealed only a 335 kDa protein upon immunoprecipitation with anti-MGAM antibodies (Naim et al., 1988a). As with SI (Naim et al., 1988a), *de novo* synthesized MGAM in biosynthetically-labeled biopsy specimens is not exposed to pancreatic enzymes, such as trypsin, and therefore it is not cleaved, in contrast to human BBMs.

Having assessed the quality of the BBMs, we performed co-immunoprecipitation experiments of SI and MGAM to determine whether an interaction occurs under different solubilization procedures and pH values and, if any, is affected by the conditions applied. Figure 2A shows that immunoprecipitations of BBM lysates with anti-MGAM (HBB 4/46) and Western blotting with anti-SUC (HBB 2/614) or anti-IM (HBB 3/705) detected heavy bands corresponding to these two proteins concomitant with a substantial interaction between SUC, IM and MGAM. The reciprocal experiment (Figure 2B), i.e., immunoprecipitation with anti-SI, pulled down a protein band of 335 kDa and also the 180 kDa form, both of which were strongly recognized in Western blots by anti-MGAM antibodies. Interestingly, the interacting 180 kDa protein band was more intensive than its counterpart in MGAM isolated with its own antibody pointing to an interaction occurring mainly between the cleaved forms of SI and MGAM.

**FIGURE 3:**
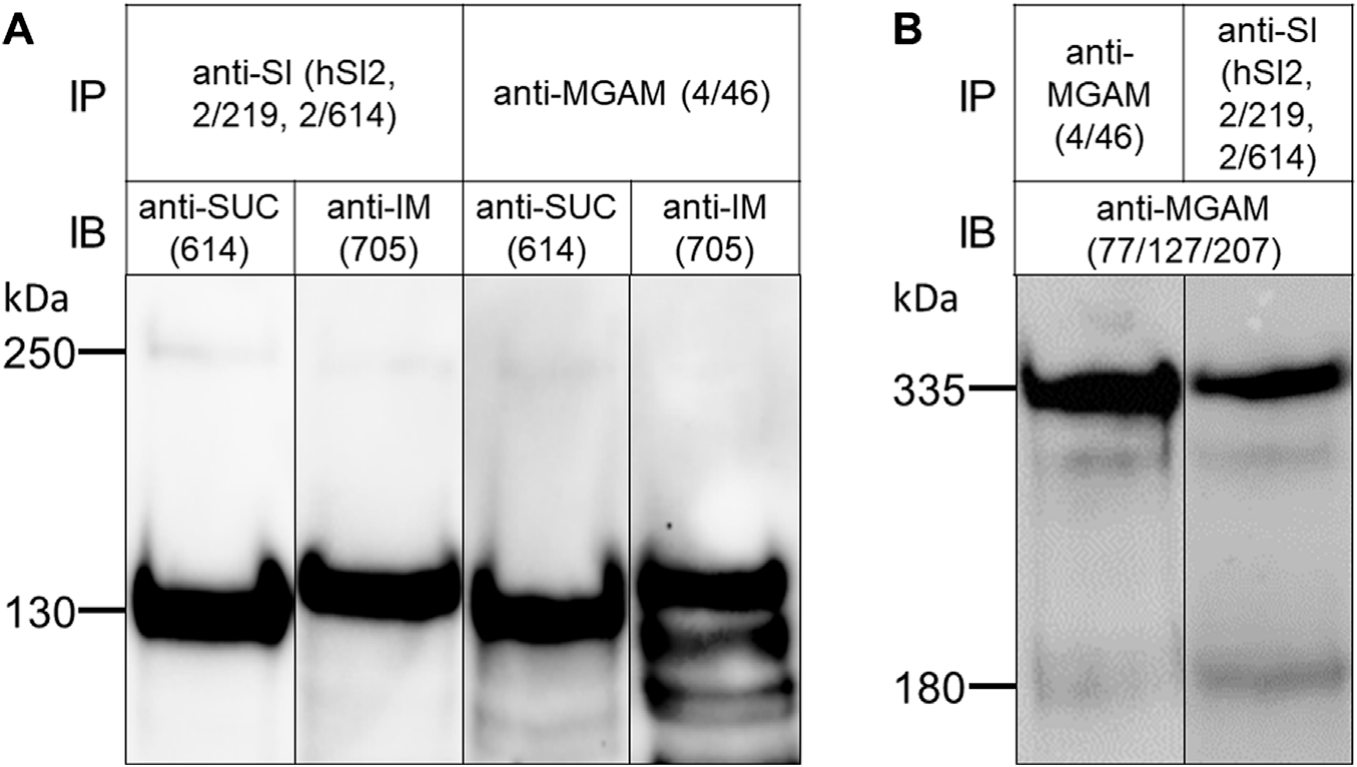
The interaction of SI and MGAM proteins is sustained in BBMs solubilized with DOC/TX-100 buffer. **(A)** BBMs were solubilized with DOC/TX-100 buffer as described in the material and methods and processed for immunoprecipitation as described in Figure 2. Similar to Figure 2A, Western blot revealed co-immunoprecipitated SUC and IM with MGAM as shown in the two right lanes. The two left lanes revealed immunoprecipitated SUC and IM with their specific antibodies comparable with the proteins in their neighboring co-immunoprecipitation lanes. **(B)** BBMs were solubilized as described in **(A)** and the immunoprecipitates of the two studied disaccharidases were analyzed by Western blot. Similar to Figure 2B, immunoblotting revealed co-immunoprecipitated MGAM protein in the right lane while the left lane contained immunoprecipitated MGAM control. SUC and IM were recognized by HBB 2/614 (anti-SUC) and HBB 3/705 (anti-IM) respectively and MGAM was recognized by anti-MGAM antibodies (77/127/207). IP, immunoprecipitation; IB, immunoblotting.

Non-ionic mild detergents such as TX-100 or lauryl-maltoside have been frequently used to characterize non-covalent protein-protein interactions, such as those existing in homo- or heterodimeric complexes. We wanted to determine whether the SI-MGAM interaction can be affected or disrupted if BBM solubilization has been performed under increased ionic and alkaline environment. Figure 3 shows that solubilization of the human BBMs with a combination of DOC/TX-100 at pH 8.0 revealed a protein band pattern of immunoprecipitated SUC, IM and MGAM similar to those obtained under non-ionic detergent conditions (see Figure 2). We therefore carried out the co-immunoprecipitations under these altered conditions as described above. Figure 3 demonstrates that also in the presence of DOC and at alkaline pH the binding capacities of SI to MGAM or *vice versa* were maintained and these enzymes were also pulled down from DOC/TX-100 lysates.

### 3.3 Interaction of SI and MGAM affects their functional capacities

**FIGURE 4:**
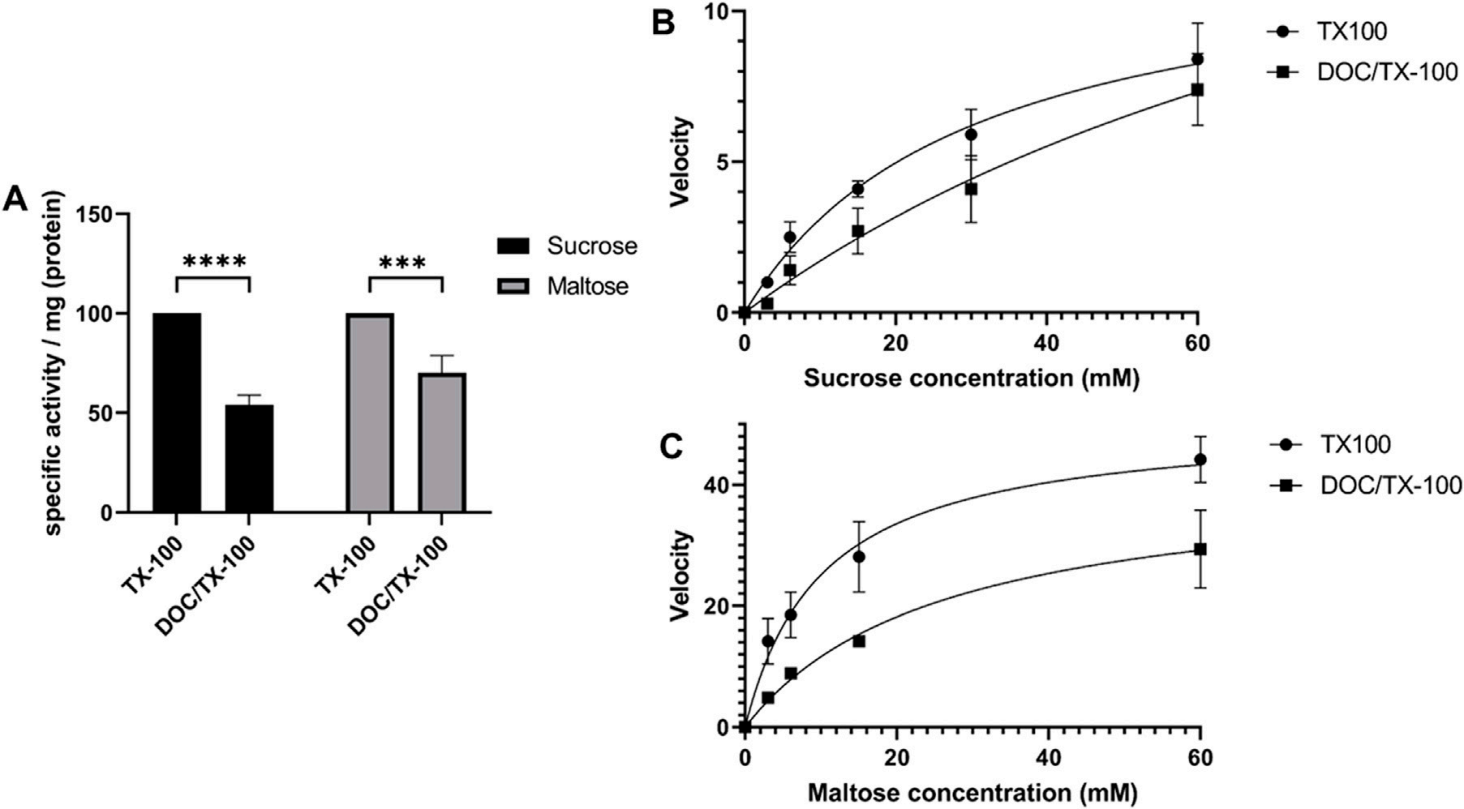
The activity and kinetics of the interacting disaccharidases are affected by detergents in the solubilization buffer used. **(A)** Specific enzymatic activity towards sucrose and maltose in human BBMs that have been solubilized with TX-100 buffer or DOC/TX-100 buffer. Lysates were incubated with 56 mM of sucrose or 56 mM of maltose as described in the materials and methods section followed by incubation with glucose fluid (GOD-PAP) to assess the liberated glucose. The results demonstrate significant variations in the enzymatic activities of SI and MGAM depending on the detergent used. * *p* < 0.05, ** *p* < 0.005 and *** *p* < 0.0005. **(B)** and **(C)** Kinetics activity measurements of SI and MGAM in BBMs solubilized with either TX-100 or DOC/TX-100 buffers. Incubation of the lysates with different concentrations of sucrose **(B)** or maltose **(C)** substrates was followed by specific enzymatic activity measurements as described before. Through Michaelis-Menten analyses, the K_m_ was determined and the results showed a lower K_m_ for both sucrose and maltose in the presence of TX-100 as compared to DOC/TX-100. The results were analyzed through GraphPad Prism’s Non-linear regression (curve fit), panel of enzyme kinetics equations, Michaelis-Menten enzyme kinetics.

In a previous study, it was shown that the specific activity of SI towards sucrose and maltose in native non-solubilized BBMs is higher than their counterparts that have been immunoprecipitated from DOC/TX-100-solubilized membranes (Amiri and Naim, 2017). This result suggested that the use of DOC in the solubilization buffer may have affected the quaternary structure of SI and its association with the membrane, particularly lipid rafts. Therefore, a comparative study comparing the activities in BBM lysates that have been extracted with DOC/TX-100 or with TX-100 alone was performed. Figure 4A shows that the specific activities contained in BBMs solubilized with DOC/TX-100 were decreased as compared to BBMs solubilized with TX-100. Similarly, there has been a significant decrease in the activity towards maltose. These results were further supported by the enzyme kinetics assessment of SUC using sucrose and maltose as substrates, which revealed that the Michaelis constant (K_m_) for both substrates was lower in the presence of TX-100 alone (Sucrose: K_m_ ≈ 30, Maltose: K_m_ ≈ 10) as compared to DOC/TX-100 (Sucrose: K_m_ ≈ 114, Maltose: K_m_ ≈ 26) (Figures 4B,C). It is possible that partial disruption of the membrane milieu, such as lipid rafts, with the combination DOC/TX-100 has resulted in the enzymatic reductions. One potential explanation correlates the size of the micelles of TX-100 and DOC/TX-100 and variations in the number of SI dimers and SI/MGAM hetero-complexes that could be accommodated within these complexes. This hypothesis is supported by the fact that micelles of TX-100 are several folds larger than those of DOC (Hasko Paradies, 1980; Esposito et al., 1987) and expectedly also than DOC/TX-100 micelles. It can be therefore postulated that DOC/TX-100 micelles accommodate a lower number of SI dimers or SI/MGAM complexes with subsequent implications on the enzymatic activities.

## 4 Discussion

The current study demonstrates an interaction between the two glycosidases SI and MGAM in detergent extracts of human intestinal BBMs. This interaction is maintained regardless of the various solubilization procedures or the pH conditions that have been utilized. Whether TX-100 has been exclusively used as a non-ionic detergent, which is expected to retain protein-protein interactions, or in combination with the slightly ionic detergent DOC, SI and MGAM interact avidly under the experimental conditions utilized. By virtue of the striking structural and functional similarities (Sim et al., 2010), an interaction occurring *via* the luminal parts resulting in heterodimeric complexes could be postulated. Another potential interaction mechanism may occur *via* the transmembrane domains of these two type II membrane glycoproteins. This mechanism would be similar to that described for type II glucosyltransferases of the Golgi and referred to as kin recognition, which occurs *via* the transmembrane domains and leads ultimately to the retention of these transferases in the Golgi (Machamer, 1991; Nilsson et al., 1993). SI has been shown to form dimers and higher order oligomers after complex glycosylation in the Golgi (Gericke et al., 2017a). These mature forms are trafficked *via* lipid rafts enriched vesicles and sorted with high fidelity to the apical membrane in Caco-2 cells (Alfalah et al., 1999; Jacob et al., 2000a). Lipid rafts therefore impact the digestive capacities of SI at the cell surface, since association of SI with lipid rafts elevates the SI function substantially (Wetzel et al., 2009) and warrants an efficient sorting to and thus full access to the disaccharides at the apical membrane. One potential mechanism that explains the elevated digestive function of lipid rafts-associated SI is that the assembly of SI in dimeric and higher order oligomers at the cell surface elicits a toggling effect within these SI clusters that enhances the digestion of the substrates by sucrase and isomaltase. A similar mechanism has been suggested for the functional performance of SI and also MGAM in a comprehensive analysis of the enzymatic activities of the individual subunits of SI and MGAM (Lee et al., 2012).

The interaction of MGAM with SI is compatible with the existence of hetero-complexes of SI and MGAM at the cell surface that may enhance the digestive function *via* a toggling mechanism within these hetero-complexes. The fact that these two glycoproteins share common hydrolytic activity towards α-1,4 glycosidic linkage in maltose (Ao et al., 2007; Lin et al., 2012) supports a mechanism implicating an interaction of both enzymes in the final steps of starch digestion. In fact, the enzymatic activity of sucrase that has been immunoprecipitated from solubilized BBM lysates towards maltose is almost two folds lower than its activity in non-solubilized BBMs, i.e., when both SI and MGAM enzymes are associated with the membrane (Amiri and Naim, 2017). This toggling concept can be further favored by the results, which revealed a decrease in SI and MGAM activity towards sucrose and maltose when a slightly ionized detergent (DOC/TX-100) was used. It can be therefore hypothesized that partial or complete disruption of these microdomains, for instance by DOC, can alter the quaternary structure of SI, MGAM and the interacting complexes SI/MGAM with subsequent reduction of the enzymatic activities. Variations in the size and shape of TX-100 and DOC/TX-100 micelles (Hasko Paradies, 1980; Esposito et al., 1987) support the hypothesis that less SI dimers and SI/MGAM hetero-complexes are accommodated within DOC/TX-100 micelles as compared to TX-100 micelles compatible with reduced activities.

Importantly, the high expression level of SI in BBMs, which is approximately 3-fold higher than that of MGAM (Amiri and Naim, 2017), indicates that the intestinal digestive function towards maltose is primarily exerted by SI and the role of MGAM is secondary to SI. In gastrointestinal disorders, such as congenital sucrase-isomaltase deficiency (CSID), when SI activities are reduced or absent, MGAM can act in an auxiliary mode partially substituting for the abolished SI function (Amiri and Naim, 2017; Husein et al., 2020).

What is the influence of potential SI and MGAM interaction on their individual trafficking behavior and in functional gastrointestinal disorders (FGID), such as irritable bowel syndrome and in CSID? CSID is restricted to variants in SI that are associated with impaired trafficking and functional deficits (Naim et al., 2012; Husein et al., 2020). It can be assumed that SI variants in CSID that are blocked intracellularly, or that are trafficked to the Golgi and blocked in that organelle may retain also MGAM in these organelles complying with a potential kin recognition mechanism (Husein et al., 2019). This retention would reduce the overall activity of MGAM at the cell surface not only because SI contributes to the overall maltose digesting capacity, but also due to impaired trafficking of MGAM that interacts with misfolded or transport-incompetent SI. Similarly, several mutations in the *SI gene* in FGID elicit variations in the trafficking pattern of SI that vary from a block in the ER to a reduced rate of maturation in the Golgi (Husein et al., 2020; Husein and Naim, 2020). In these cases, an interaction between SI and MGAM might also result in a similar trafficking pattern for MGAM with eventual negative implications on the overall maltose digestive capacities at the cell surface. Molecular analyses in intestinal cells that express variants of SI in conjunction with wild type MGAM are required to verify this mechanism.

## Data Availability

The raw data supporting the conclusions of this article will be made available by the authors, without undue reservation.
